# Differences in lung cancer characteristics and mortality rate between screened and non-screened cohorts

**DOI:** 10.1038/s41598-019-56025-6

**Published:** 2019-12-18

**Authors:** Fu-Zong Wu, Pei-Lun Kuo, Yi-Luan Huang, En-Kuei Tang, Chi-Shen Chen, Ming-Ting Wu, Yun-Pei Lin

**Affiliations:** 1 Department of Radiology, Kaohsiung Veterans General Hospital, Kaohsiung, Taiwan; 2 Faculty of Medicine, School of Medicine, National Yang Ming University, Taipei, Taiwan; 3 Institute of Clinical Medicine, National Yang Ming University, Taipei, Taiwan; 4 Department of Surgery, Kaohsiung Veterans General Hospital, Kaohsiung, Taiwan; 5 Department of Nursing, Shu-Zen Junior College of Medicine and Management, Kaohsiung, Taiwan; 6 Physical Examination Center, Kaohsiung Veterans General Hospital, Kaohsiung, Taiwan

**Keywords:** Oncology, Cancer, Cancer screening

## Abstract

Screening programs for lung cancer aim to allow diagnosis at the early stage, and therefore the decline in mortality rates. Thus, the aim of this retrospective cohort study was to the comparison of screened and non-screened lung cancer in terms of lung cancer characteristics, overdiagnosis and survival rate. A retrospective study in which 2883 patients with 2883 lung cancer diagnosed according to the hospital-based lung cancer register database between 2007 and 2017. A comparison was performed in term of clinical characteristics and outcomes of lung cancer between the screened and non-screening patient groups. 2883 subjects were identified (93 screened and 2790 non-screened). Screened group patients were younger (59.91 ± 8.14 versus 67.58 ± 12.95; p < 0.0001), and were more likely to be female than non-screened group (61.3% versus 36.8%; p < 0.0001). The screened group showed significantly better outcomes in overall mortality than the non-screened group (10.75% versus 79.06%; <0.0001). In a Cox proportional hazard model, lung cancer in the screened group proved to be an independent prognostic factor in lung cancer subjects. Our findings point to the improved survival outcome in the screened group and might underline the benefit of low-dose computed tomography (LDCT) screening program in Asian populations with the high prevalence of non–smoking-related lung cancer. Further study aimed at the LDCT mass screening program targeting at light smokers and non-smoker outside of existing screening criteria is warranted.

## Introduction

In Taiwan, lung cancer is the leading cause of cancer-related deaths and among both men and women over the last decade. 5884 males and 3348 females died of lung cancer in 2015, accounting for the most common cancer-related mortality rate among 10 most common cancers according to the Taiwan Cancer Registry annual report.

The National Lung Screening Trial (NLST) demonstrated that lung cancer screening using low dose computed tomography (LDCT) resulted in a significant reduction in lung cancer-specific overall mortality rate in comparison with chest radiography in high-risk heavy smokers^1^. Around 70~80% of patients with lung cancer diagnosed have stage III or IV disease at presentation in Taiwan, which generally lead to poor prognosis with the high mortality rate^2^. Therefore it is crucial that screening and diagnosis of lung cancer at early stage because when found early, lung cancer is highly curable^3^.

Previous studies have demonstrated that family history and gender are two important factors associated with non-smoking related lung cancer^4,5^. In addition, the average PM2.5 levels in Taiwan were much higher than the levels in European cities according to the European Study of Cohorts for Air pollution Effects (ESCAPE), especially in Kaohsiung city^6^. Therefore, genetic factors and environmental pollution may play an important role in the development of non-smoking related lung cancer. Therefore, the LDCT screening selection criteria should be modified due to regional genetic and environmental differences. In Kaohsiung Veterans General Hospital (Kaohsiung, Taiwan), self-paid LDCT for lung cancer screening has been conducted for population-based mass screening aged 40~80 years since 2007 in a population with the high prevalence of non-smoking-related lung cancer^4,7^. However, all screening programs involve the trade-offs between potential benefits and potential harms. There are still many unresolved problems about the benefits, harms and the cost of the mass screening, included light smokers and non-smokers^8–11^.

Thus, the aim of this retrospective cohort study was to a comparison of screened and non-screened lung cancer in terms of lung cancer characteristics, overdiagnosis and survival rate.

## Materials and Methods

Between January 2007 and September 2017, 2883 patients were diagnosed with lung cancer at Kaohsiung Veterans General Hospital. The focus in this present study is on clinical characteristics and outcomes of lung cancer between screened and non-screening patient groups. The institutional review board approved this retrospective study, and thus informed consent was waived. All research was performed in accordance with the relevant guidelines and regulations and all Institutional Review Board requirements.

### Definition of screened and non-screened lung cancer

Patients were classified two groups: Group 1 (screened group): patients who had no clinical symptoms and incidentally detected lung cancers by self-paid LDCT exam aged 40–80 years. Group 2 (non-screened group): patients with the clinical scenario of lung cancer-related symptoms or abnormal finding (highly suspicion of lung cancer) in the chest radiograph were further diagnosed as lung cancer. Patients were assigned to screened and non-screened subgroups based on the stringent definition. Our database included the following clinical characters: age, sex, tumor size, histopathologic type, adenocarcinoma spectrum classification, lung cancer clinical stage, death, survival time, mortality rate, smoking habit, betel nut and alcohol drinking habit. The histological diagnosis was described according to the World Health Organization classification. All the patients were staged according to the 7th edition of the TNM staging system published in 2009^12^. Data regarding curative surgical treatment and targeted therapy use were also recorded.

### Statistics

All statistical analyses were performed with SPSS 17.0 for Windows (SPSS Inc, Chicago, IL), STATA 13.0 package (StataCorp, College Station, TX, USA) and MedCalc 13.2.2.0 (MedCalc Software, Ostend, Belgium). Differences in continuous variables between 2 groups were compared by the independent Student t test. Categorical variables were summarized as frequencies and percentages and compared using the chi-square test to examine differences in demographic characteristics. Fisher’s exact test was used to analyze when the smallest expected value is less than 5. Overall survival time was defined as the period from the date of lung cancer diagnosed until the date of death from any cause or until the date of the last follow-up at which point the data were censored. Overall survival was estimated using the Kaplan-Meier method. A log rank test was used to compare prognosis between screened and non-screening groups. Cox regression analysis was performed to calculate and compare the survival rate. A multivariate analysis was used to estimate the hazard ratios (HRs) based on the Cox regression model, adjusted for age, gender, smoking, alcohol, betel nut, tumor size, curative surgery, target therapy, histology and screened or not. A p value of 0.05 was regarded as significant.

### Ethics approval

The study protocol was approved by the Institutional Review Board of Kaohsiung Veterans General Hospital (VGHKS19-CT2–09). The need for informed consent was waived by the institutional review board due to the minimal risk retrospective study.

## Results

### Baseline characteristics

During the study period, 2883 lung cancer patients were analyzed according to the hospital-based cancer registry dataset. The screened group consisted of 93 subjects, and the non-screened group consisted of 2790 subjects. The general characteristics of the lung cancer subjects between the two groups are summarized in Table 1.

**Table 1:** Clinical characteristics of 2883 lung cancer patients diagnosed in a hospital-based cohort according to screened status.

	Screened (N = 93)	Non-screened (N = 2790)	P- value
Mean age at diagnosis, years (mean, SD)	59.91 ± 8.14	67.58 ± 12.95	<0.0001
Median age at diagnosis	60 (43–75)	69 (40–79)	<0.0001
Gender (n, %)			<0.0001*
Male	36 (38.7%)	1764 (63.2%)	
Female	57 (61.3%)	1026 (36.7%)	
Smoking	12 (12.9%)	1220 (43.7%)	<0.0001*
Alcohol drinking	10 (10.8%)	444 (15.9%)	0.196*
Betel nut	3 (3.2%)	195 (7%)	0.209**
Histology			<0.0001**
Adenocarcinoma	88 (94.6%)	2013 (72.1%)	
Squamous cell carcinoma	3 (3.2%)	444 (15.9%)	
Small cell carcinoma	1 (1.1%)	257 (9.2%)	
Other	1 (1.1%)	77 (2.8%)	
Adenocarcinoma spectrum			<0.0001**
AAH	6 (6.8%)	0	
AIS	7 (7.9%)	4 (0.2%)	
MIA	9 (10.2%)	0	
IPA	66 (75%)	2009 (99.8%)	
Stage			<0.0001**
Carcinoma *in situ*	14 (15.0%)	4 (0.14%)	
I	62 (66.7%)	455 (16.31%)	
II	5 (5.3%)	124 (4.44%)	
III	4 (4.3%)	636 (22.80%)	
IV	8 (8.6%)	1571 (56.31%)	
Curative surgery rate	82 (88.17%)	670 (24.01%)	<0.0001*
Target therapy	6 (6.45%)	530 (18.99%)	<0.0001*
Mean tumor size (mm)	17.52 ± 14.98	45.53 ± 25.29	<0.0001
Death	10 (10.75%)	2206 (79.06%)	<0.0001*
Mean survival days	860.31 ± 502.20	574.94 ± 558.48	<0.0001
Median survival days	793 (22–2599)	404 (1–3128)	<0.0001

Abbreviations: AAH: atypical adenomatous hyperplasia; AIS: adenocarcinoma *in situ*; MIA: minimally invasive adenocarcinoma; IPA: invasive pulmonary adenocarcinoma; SD: standard deviation.

*Using Chi-square test for categorical variables.

**Using Fisher’s exact test for categorical variables.

The mean age in the screened group was significantly younger than that in the non-screened group (59.91 ± 8.14 years old versus 67.58 ± 12.95 years old, p < 0.0001; median age 60 years-old versus 69 years-old). The screened patients had a significantly greater proportion of female gender than the non-screened group (61.3% versus 36.8%; p < 0.0001). Patients in the non-screening group had the significantly higher proportion of smoking habit (p < 0.0001), surgery (p = 0.0001) and target therapy (p = 0.001) than the screened group. There were no significant differences between the two groups in terms of alcohol drinking and betel nut habits. For survival interval analysis, the mean survival date in the screened group was significantly higher than that in the non-screened group (860.31 ± 502.20 versus 574.94 ± 558.48 days; median survival days 793 versus 404, p < 0.0001).

### Comparison of histopathology and clinical lung cancer stage distribution

The comparison of the difference in histopathology and clinical lung cancer stage distribution between the two groups are summarized in Table 1. A significant difference in stage at diagnosis was seen between screened and non-screened groups.

Stage I was the most frequent stage in the patients with screened group. In comparison, patient in the non-screened group presented with stage IV in most cases.

For carcinoma *in situ*, the screened patients had a significantly greater proportion of carcinoma *in situ* than the non-screened group (15% vs. 0.14%; p < 0.0001).

For lung cancer histopathology distribution, a significant difference in histopathology distribution was seen between screened and non-screened groups. With regard to histological subtype, adenocarcinoma was significantly more prevalent in the patients with screened group (94.62% vs. 72.15%; p < 0.0001). In contrast to that, the subjects with non-screened group have a significantly greater proportion of the patients with small cell carcinoma (15.91% vs. 3.22%; p < 0.0001) and squamous cell carcinoma (9.20% vs. 1.07%; p < 0.0001). With regard to histological subtype of adenocarcinoma spectrum, a significant difference in histological subtype of adenocarcinoma spectrum was seen between the screened and non-screened groups. Invasive pulmonary adenocarcinoma was the most frequent histological subtype in the patients with non-screened group. In comparison, the patients in screened group presented with higher portion of minimally invasive adenocarcinoma, adenocarcinoma *in situ* or its precursor lesions.

Table 2 summarizes the mortality and survival analysis according to screened and non-screened groups in term of overall, 1-year and 5-year mortality rates.

**Table 2:** Mortality and survival profiles of lung cancer patients diagnosed in a hospital-based cohort according to screened status.

	Screened group	Non-screened group	P- value
Patient	N = 93	N = 2790	
Death number	N = 10	N = 2206	
1 year mortality	2.66%	44.63%	<0.0001
5 year mortality	31.42%	88.00%	<0.0001
Overall mortality	10.75%	79.06%	<0.0001
Average survival days	860.31 ± 502.20	574.94 ± 558.48	<0.0001

Patients in the screened group had a lower overall mortality than that in the non-screened group. One-, and Five-year mortality rates increased significantly from the screened group to the non-screened group (*P* < 0.0001 for all). The mean survival time for the screened group was 860.31 ± 502.20 days (median 793 days), and the mean survival for the non-screened group was 574.94 ± 558.48 days (median 404 days). After exclusion of AAH or AAH/AIS patients, the screened group still showed significantly better outcomes in overall mortality than the non-screened group shown in Supplement Tables 1 and 2.

### Association of clinical prognostic variables with survival

**Figure 1:**
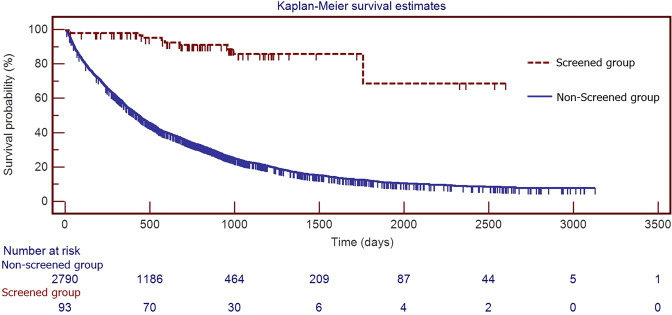
Kaplan-Meier survival analysis in screened and non-screened groups of lung cancer patients. The survival curves differed significantly (by log-rank test, P = 0.0001).

Survival analysis for lung cancer using Cox regression model for multivariate effects, the hazard ratio for lung cancer mortality was determined adjusting for age, gender, smoking, alcohol, betel nut and screened status (Table 3). Multivariate survival analysis using Cox’s regression model showed age (HR = 1.017, P = 0.001), gender (HR = 0.824, P = 0.0001), smoking status (HR = 1.281, P = 0.0001), screened status (HR = 0.329, P = 0.0001), target therapy (HR = 1.009, P = 0.0001), curative surgery (HR = 0.348, P = 0.0001), Tumor size (HR = 1.000304, P = 0.009), and histologic type (HR = 0.790, P = 0.0001) were as identified independent prognostic factors of overall survival for lung cancer patients shown in Table 3. Survival times were determined as of May 2018. During this period, 2206 patients (79.1%) died, and 584 patients (20.9%) were censored in the non-screened group. In addition, 10 patients (10.8%) died, and 83 patients (89.2%) were censored in the screened group. Comparing survival curves of two groups using the log rank test, survival was significantly superior in the patients with screened group compared with the patients in non-screened group shown in Fig. 1 (P = 0.0001).

**Table 3:** Multivariate Cox regression analysis of prognostic factors of patients with lung cancer diagnosed in a hospital-based cohort.

Variable	Hazard Ratio	95% CI	P- value
Age	1.017	1.014–1.021	0.001
Gender	0.824	0.735–0.924	0.0001
Smoking	1.281	1.147–1.430	0.0001
Alcohol	1.041	0.903–1.199	0.582
Betel nut	1.001	0.822–1.218	0.995
Screened	0.329	0.176–0.615	0.0001
Tumor size	1.000304	1.000188–1.000419	0.009
Target therapy	1.009	1.002–1.015	0.0001
Histology	0.790	0.719–0.869	0.0001
Curative surgery	0.348	0.311–0.391	0.0001

Abbreviations: CI: confidence interval.

Histology: adenocarcinoma versus other histology type (reference).

## Discussion

The focus of the present study is to compare between screened and non-screened lung cancer in terms of lung cancer characteristics, overdiagnosis and survival rate. In this study, we demonstrated four major findings. The first one is that we observed that adenocarcinoma was significantly more frequent in patients in the screened group. The second finding is that stage I was significantly more frequent in patients in the screened group. Third, favorable overall survival was significantly superior in the screened group and lead to a reduction of 68.31% compared with non-screened group.

Fourth, there was an obvious increase of carcinoma *in situ* lesions in the screened group and lead to an increase of 14.86% compared with the non-screened group in regard to overdiagnosis.

In 2011, the NLST reported a reduction in lung cancer mortality of 20% in subjects who underwent annual LDCT exam compared with annual chest x-ray exam during the time period of 5 years^1^. In this present study, we found a significant decrease in the overall mortality rate of 68.31% in the screened group compared with the non-screened group. Our present result is similar to the findings of this previous study^1^. Further, in accordance with our results, lung cancer subjects in the screened group also had smaller primary tumor size and lower clinical stage at the time of initial diagnosis. These finding were associated with the lower mortality rate, longer survival periods, and an increased surgical treatment rate. However, there are also a number of important differences between the NLST population and our screened group. Our screening population found a higher proportion of non-smoking women with lung cancer diagnosed. These findings also support that there appears to be an increasing trend of non-smokers related lung cancer in Asian population such as China, Japan, Korea and Taiwan in recent years^4,7^. Currently most of the society recommends that lung cancer screening targeted in adults age 55 to 80 year who have a 30 pack-year smoking history and currently smoke or have quitted within the past 15 years^11,13,14^.

The present results also support previous studies concerning broadly expanding inclusion criteria to include never-smokers in lung cancer screening program in Asian population with the high prevalence of non-smoking related lung cancer^4,5,7,8,15^. Recent research has demonstrated that lung cancer screening using LDCT resulted in a 43% decrease in all-cause death in comparison with chest radiography in a population-based cohort including non-smoker and light smoker in Hitachi city, Japan^15,16^. Therefore, the effectiveness of lung cancer screening program specifically targeting at non-smoking Asian population with the high prevalence of non-smoking related lung cancer should be further investigated.

### The issue of overdiagnosis and overmanagement

In this study, we assessed the factors affecting the prognosis of lung cancer between the screened and non-screened groups. In the multivariable Cox regression model, lung cancer in the screened group is the most important factor responsible for improved survival prognosis. Therefore, it is crucial that widely implementation of LDCT lung cancer screening program in Asian population including non-smoker and light smoker based on the lung cancer risk prediction model. However, overdiagnosis is always an important concern in cancer screening because it potentially leads to overtreatment and potential harmful treatments^17,18^. In this study, we attempted to estimate the proportion of carcinoma *in situ* or the proportion of early adenocarcinoma spectrum lesions to assess the extent of overdiagnosis. For carcinoma *in situ*, the screened patients had a significantly greater proportion of carcinoma *in situ* than the non-screened group (15% vs. 0.14% p < 0.0001). For adenocarcinoma lesions, the screened patients had a significantly greater proportion of precursor lesions of adenocarcinoma spectrum (including AAH, AIS, and MIA) than the non-screened group (23.65% vs. 0.19% p < 0.0001). To estimate the percentage of overdiagnosis in NLST trial, Patz *et* al. estimated that more than 18% of all lung cancers detected by LDCT in the NLST seem to be indolent^17^. Our findings also support the same results.

There is even a more obvious trend because of the higher prevalence of non-smoking lung adenocarcinoma spectrum lesions in Asia^4,7,19,20^. Recent evidences have demonstrated that a strong correlation between radiologic features and adenocarcinoma spectrum lesions^21,22^. In addition to the rapid development of computer image processing and radiomics technology, recent studies have also demonstrated that use of CT radiomic texture analysis such as tumor size, morphology, attenuation, entropy and volumetric doubling time analysis could facilitate more accurate differentiation between pre-invasive lesions and invasive pulmonary adenocarcinomas^23–29^. Our previous studies also have shown that novel subclassification of subsolid nodules has the high positive predictive value to rule in invasive pulmonary adenocarcinoma, which manifesting as part-solid nodules^23,24^. Incorporation of these CT radiomic features can help further refine the management of subsolid nodules in the Asian lung cancer screening program with the high prevalence of non-smoking related lung cancer^4,7,30^. The goal of lung cancer screening program is to reduce mortality rate from lung cancer death and avoid overdiagnosis/overmangement. Thus, we should always be aware of overdiagnosis as a counterpart of lung cancer screening program in Asia. Because of more indolent growth for preinvasive lesions which manifesting as pure groundglass nodules, the combination strategies of shared decision making and watchful waiting with annual or biannual follow-up for a period of at least 5 years a would be a safe option to allow early detection of disease progression and to avoid the potential harms of overdiagnosis and overtreatment^25,31,32^. These strategies could improve the efficacy of lung cancer screening programs in Asian populations with the high prevalence of non–smoking-related lung cancer^7,25^.

### Strength and limitations

The main strength of this study is the hospital-based lung cancer registry database, which mainly reflect the entire lung cancer population in a hospital-based cohort. Therefore, the large hospital-based cohort could result in sufficient power to detect small differences in patient outcomes and prognosis. Several previous studies investigated the difference between screened and symptomatic group for surgically diagnosed lung cancer subjects, which can’t represent the entire distribution of lung cancer in the population^33,34^.

This study also has its limitations. First, this study is a retrospective cohort study design. Therefore, the retrospective aspect may introduce selection bias and information bias, which could bias this study’s results. Second, it is uncertain that the current study results could be widely generalized to other hospitals in Asia. Thus further large multicenter studies are needed to investigate the effect of implementation of mass lung cancer screening program at target-risk population based on lung cancer-risk prediction model to establish the generalizability in Asia^9,30^.

## Conclusions

In summary, a very high proportion of non-smoking related lung adenocarcinoma is detected at the early stage in patient underwent mass lung cancer screening program, which lead to significantly improved survival outcome. These results suggest that broadly expanding inclusion criteria to include never-smokers in lung cancer screening program in Asian population may be crucial in reducing mortality rate and improving survival outcome. To maximize the cost-effectiveness and health benefits of LDCT screening and reduce the potential risk in over-diagnosis and over-management among non-smokers, further prospective-cohort studies should address the effectiveness of implementation of LDCT lung cancer screening program targeting at high-risk population based on lung cancer prediction risk model.

## Supplementary information

Supplement Table 1&2

## Data Availability

The datasets used and/or analyzed during the current study are available from the corresponding author on reasonable request.
