# Author Correction: Differences in lung cancer characteristics and mortality rate between screened and non-screened cohorts

**DOI:** 10.1038/s41598-021-98096-4

**Published:** 2021-09-16

**Authors:** Fu-Zong Wu, Pei-Lun Kuo, Yi-Luan Huang, En-Kuei Tang, Chi-Shen Chen, Ming-Ting Wu, Yun-Pei Lin

**Affiliations:** 1 Department of Radiology, Kaohsiung Veterans General Hospital, Kaohsiung, Taiwan; 2 Faculty of Medicine, School of Medicine, National Yang Ming University, Taipei, Taiwan; 3 Institute of Clinical Medicine, National Yang Ming University, Taipei, Taiwan; 4 Department of Surgery, Kaohsiung Veterans General Hospital, Kaohsiung, Taiwan; 5 Department of Nursing, Shu-Zen Junior College of Medicine and Management, Kaohsiung, Taiwan; 6 Physical Examination Center, Kaohsiung Veterans General Hospital, Kaohsiung, Taiwan

Correction to: *Scientific Reports* 10.1038/s41598-019-56025-6, published online 18 December 2019

In the original version of this Article, Fu-Zong Wu was incorrectly listed as a corresponding author. The correct corresponding author for this Article is only Yun-Pei Lin. Correspondence and request for materials should be addressed to fc760203@gmail.com.

This error has now been corrected in the HTML and PDF versions of the Article and in the accompanying Supplementary Information file.

## Supplementary Information

Supplementary Tables.

