# Supplementary material for: Differences in lung cancer characteristics and mortality rate between screened and non-screened cohorts
Source: Sci Rep. 2019 Dec 18;9:19386. doi: 10.1038/s41598-019-56025-6 (PMC6920422; doi:10.1038/s41598-019-56025-6)
Supplement: Supplementary file 1 — Supplement Table 1&2 [file 41598_2019_56025_MOESM1_ESM.docx]

**Title Page**

**Title**

Differences in lung cancer characteristics and mortality rate between screened and non-screened cohorts.

**Authors:**

Fu-Zong Wu, MD^1,2,3^, Pei-Lun Kuo, MD, PhD^2,3^ ,Yi-Luan Huang, MD^1,2,3^, En-Kuei Tang, MD^4,5^, [Chi-Shen Chen](javascript:void(0);), MD^6^, Ming-Ting Wu, MD^1,2,3^,Yun-Pei Lin, MD^1,2,3,*^

**Author Affiliations:**

^1^Department of Radiology, Kaohsiung Veterans General Hospital, Kaohsiung, Taiwan

^2^Faculty of Medicine, School of Medicine, National Yang Ming University, Taipei, Taiwan

^3^Institute of Clinical Medicine, National Yang Ming University, Taipei, Taiwan

^4^Department of Surgery, Kaohsiung Veterans General Hospital, Kaohsiung, Taiwan

^5^Department of Nursing, Shu-Zen Junior College of Medicine and Management, Kaohsiung, Taiwan

^6^Physical Examination Center, Kaohsiung Veterans General Hospital, Kaohsiung, Taiwan

**Address correspondence to**:

*Yun-Pei Lin, MD, Department of Radiology, Kaohsiung Veterans General Hospital, Taiwan No.386, Ta-Chung 1st Road, Kaohsiung, Taiwan 81362

E-mail contact: fc760203@gmail.com

***Keywords***

LDCT; Lung cancer; Screening; Mortality rate; Hospital-based cancer registry

| Supplement Table 1. Mortality and survival profiles of lung cancer patients diagnosed in a hospital-based cohort according to screened status (exclusion of AAH/AIS patients) | | | |
| --- | --- | --- | --- |
|  | Screened group | Non-screened group | P-value |
| Patient | N=79 | N=2787 |  |
| Death number | N=10 | N=2206 |  |
| 1 year mortality | 2.55% | 41.58% | <0.001 |
| 5 year mortality | 16.81% | 79.25% | <0.001 |
| Overall mortality | 11.50% | 79.10% | <0.001 |
| Average survival days | 872.56 ± 526.77 | 574.72 ± 558.74 | <0.001 |
| Abbreviations: AAH: atypical adenomatous hyperplasia; AIS: adenocarcinoma in situ | | | |

| Supplement Table 2. Mortality and survival profiles of lung cancer patients diagnosed in a hospital-based cohort according to screened status (exclusion of AAH patients) | | | |
| --- | --- | --- | --- |
|  | Screened group | Non-screened group | P-value |
| Patient | N=87 | N=2790 |  |
| Death number | N=10 | N=2206 |  |
| 1 year mortality | 2.31% | 41.53% | <0.001 |
| 5 year mortality | 15.90% | 79.16% | <0.001 |
| Overall mortality | 11.50% | 79.10% | <0.001 |
| Average survival days | 861.02 ± 513.63 | 574.94 ± 558.48 | <0.001 |
| Abbreviations: AAH: atypical adenomatous hyperplasia; AIS: adenocarcinoma in situ | | | |
